# Building a ninefold symmetrical barrel: structural dissections of centriole assembly

**DOI:** 10.1098/rsob.150082

**Published:** 2015-08-12

**Authors:** Gang Dong

**Affiliations:** Max F. Perutz Laboratories, Medical University of Vienna, Vienna 1030, Austria

**Keywords:** centriole, protein, structure

## Abstract

Centrioles are short microtubule-based organelles with a conserved ninefold symmetry. They are essential for both centrosome formation and cilium biogenesis in most eukaryotes. A core set of five centriolar proteins has been identified and their sequential recruitment to procentrioles has been established. However, structures at atomic resolution for most of the centriolar components were scarce, and the underlying molecular mechanisms for centriole assembly had been a mystery—until recently. In this review, I briefly summarize recent advancements in high-resolution structural characterization of the core centriolar components and discuss perspectives in the field.

## 1. Introduction

Centrioles are cylindrical cellular structures present in almost all eukaryotic lineages. They are 0.1–0.5 µm long and 0.1–0.2 µm in diameter, and are usually composed of nine microtubule triplets at their outer wall [1,2]. Exceptions to this organization are found in *Drosophila* embryos (nine doublet microtubules), and in *Caenorhabditis elegans* sperm cells and early embryos (nine singlets).

Centrioles usually exist in pairs, oriented perpendicularly to one another, and are surrounded by the pericentriolar material (PCM) to form the centrosome [3,4]. The PCM has a layered structure made of fibres and matrix proteins, and is responsible for microtubule nucleation and anchoring [5–8]. As such, the centrosome is the major microtubule organizing centre of animal cells. Centrosome duplication is controlled by centriole replication, which in most animal cells is tightly coordinated with the mitotic cycle. Miscoordination of the two processes often leads to alterations in centrosome number and/or structure that result in chromosome missegregation and severe consequences such as cancerous growth of the cell [9–12]. Besides nucleating centrosomes, centrioles also play a pivotal role in the biogenesis and operation of the cilium, an antenna-like structure projecting out from the cell surface [13]. Cilia are present on virtually every human cell type, and defects in their assembly or function lead to a plethora of human disorders, which are collectively called the ciliopathies [14,15].

Despite cell biologists' great interest in the centriole and extensive cellular and microscopic studies on this organelle for several decades, the molecular composition of the centriole was not fully determined until recently. Because of its favourable features for laboratory study and the availability of powerful genetic and genomic tools, the nematode *C. elegans* has become an important model organism in studying centrosome duplication and centriole assembly. Studies in several research groups have uncovered five core *C. elegans* centriolar proteins: the polo-like kinase ZYG-1 [16], and the four coiled-coil-containing proteins, SPD-2 [17,18], SAS-4 [19,20], SAS-5 [21] and SAS-6 [22,23]. All of these proteins contain both structured domains and disordered segments and are conserved in all ciliated cells.

**Figure 1.**
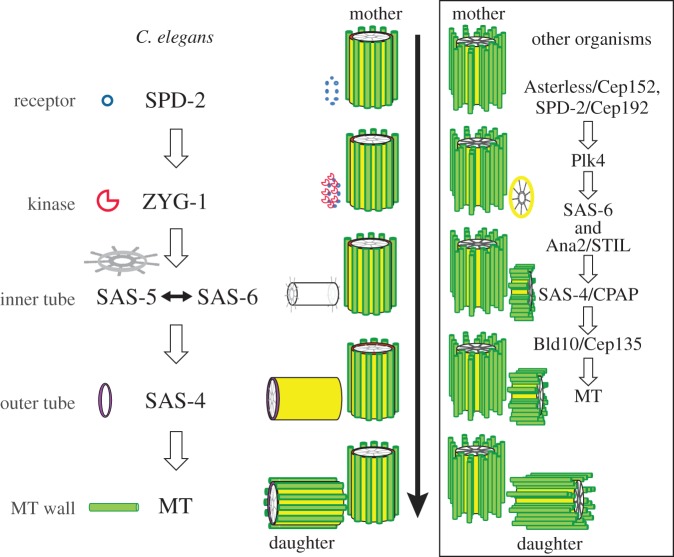
Centriole assembly in *C. elegans*. Centriole assembly is initiated by the recruitment of SPD-2 to the vicinity of the mother centriole. ZYG-1 is incorporated and then recruits the SAS-5/SAS-6 complex that forms the inner tube. Incorporation of SAS-5/SAS-6 promotes SAS-4 recruitment, which generates the outer tube. Finally, nine singlet microtubules assemble symmetrically around the outer tube to generate the microtubule wall. For comparison, a schematic of a similar stepwise centriole assembly in other organisms is shown on the right, with the major involved proteins being listed.

Centriole duplication is a multistep process, with each of the five centriolar proteins being recruited in a hierarchical manner [24,25]. In *C. elegans*, centriole assembly is initiated by the recruitment of SPD-2 to the proximal side of the mother centriole. The kinase ZYG-1 is then incorporated into the SPD-2 scaffold, and is required for the subsequent recruitment of the SAS-5/SAS-6 complex. SAS-4 is then recruited to add an outer tube around the central tube formed by SAS-5 and SAS-6. Finally, nine singlet microtubules are assembled onto the outer tube to generate a daughter centriole (figure 1). In other organisms, including *Drosophila* and humans, additional essential proteins besides the five core components are also recruited during centriole assembly (figure 1).

Recent years have seen a surge of structural dissections of the centriole, with an increasing number of high-resolution structures reported for most of its core components. Together, these have greatly advanced our understanding of centriole duplication at the atomic level. Nevertheless, a full molecular view of centriolar assembly is a mission that is not yet completed. This review briefly summarizes recent progress in the structural characterization of centriole assembly, which promises a challenging but rewarding future.

## 2. SPD-2/Cep192 and Asterless/Cep152

**Figure 2.**
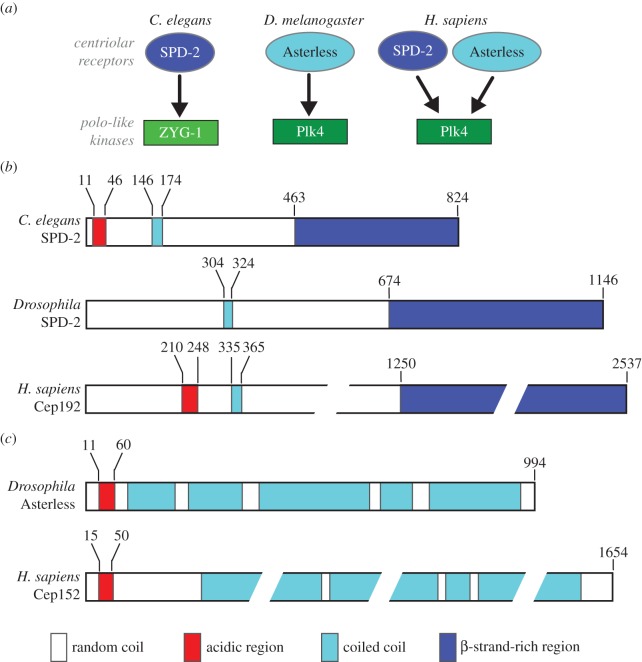
Recruitment of the polo-like kinases Plk4/ZYG-1 and domain organization of the centriolar receptors. (*a*) Different recruitment modes of ZYG-1/Plk4 by their respective centriolar receptors SPD-2/Asterless. *Caenorhabditis elegans* has only one centriolar receptor, SPD-2, to recruit ZYG-1. *Drosophila* has both SPD-2 and Asterless, but only Asterless can bind and recruit Plk4. In humans, both SPD-2 and Asterless bind Plk4 but in a mutually exclusive manner to temporally and spatially regulate Plk4 recruitment. (*b*) Domain organization of SPD-2 homologues. Residue numbers of domain boundaries are shown above the schematics. (*c*) Domain organization of Asterless and Cep152.

Centriole duplication in animals is initiated by recruiting the centriolar receptors SPD-2 and/or Asterless to the vicinity of the mother centriole. These receptors then recruit polo-like kinase 4 (Plk4) and ZYG-1, depending on which organism is under consideration (figure 2*a*). SPD-2 is a coiled-coil-containing protein localizing to both the centriole and the PCM in *C. elegans* [17,18]. It has an N-terminal acidic region that binds to the cryptic polo-box (CPB) domain of ZYG-1 (figure 2*b*) [26]. The *Drosophila* orthologue of SPD-2 lacks the acidic region for Plk4 binding, and thus is dispensable for centriole duplication [27,28] (figure 2*b*). The mammalian SPD-2 orthologue Cep192 also bears an acidic region that interacts with the Plk4 CPB domain, but it is located a bit further downstream in the sequence (figure 2*b*).

Asterless was originally identified in *Drosophila* as a protein essential for aster formation during male meiosis [29]. The protein was later revealed to be a constitutive centriolar component involved in the initiation of centriole duplication [30–33]. Cep152, the mammalian orthologue of Asterless, is also involved in both centriole duplication and PCM recruitment [34–36]. Both *Drosophila* Asterless and human Cep152 bind to the CPB domain of Plk4 via their N-terminal acidic region [32,35]. Recent studies demonstrated that Plk4 additionally binds to the C-terminal region of Asterless, which serves to stabilize Plk4 during mitosis [37].

Although it is now well established how SPD-2 and Asterless interact with their kinase partners ZYG-1 and Plk4, they have been structurally less characterized. Bioinformatics analysis suggests that all SPD-2/Cep192 orthologues contain a mostly unstructured N-terminal region with a short coiled-coil domain and a long C-terminal *β*-strand-rich region (figure 2*b*). By contrast, all Asterless/Cep152 orthologues contain mostly helical structures that are predicted to form coiled-coils, with no *β*-strand content (figure 2*c*). *Caenorhabditis elegans* and mammalian SPD-2, and *Drosophila* and mammalian Asterless, also contain a highly acidic region towards their N-termini, which interacts directly and tightly with their kinase partners [26]. This tight interaction creates something of a puzzle however, as the receptors and the kinases do not seem to form a robust complex in the cell until the receptors are targeted to mother centrioles. Recent studies by multiple imaging/biophysical techniques have shown that *C. elegans* SPD-2 is monomeric in the cytoplasm [38]. It was postulated that the acidic region for kinase binding might be concealed via its interaction with another (probably basic) part of the same protein; receptors targeted to the nascent centriole might then release such auto-inhibition to facilitate interaction with the downstream kinases [26].

Another unresolved issue about the centriolar receptors (SPD-2 and Asterless) in centriole assembly is how they themselves are recruited to the mother centriole. One possibility is that Plk1, another critical polo-like kinase linking centriole biogenesis to cell-cycle progression [39–41], might phosphorylate SPD-2/Asterless to promote their targeting and the initiation of centriole assembly. In human cells, co-localization of another coiled-coil-rich protein Cep63 with Cep152 in a ring-like structure around the proximal end of the mother centriole has been shown [42]. Cep63 knock-out leads to both Cep152 mislocalization and deficiency in procentriole assembly. It was hypothesized that Cep63 might oppose the activity of Plk1 that modifies centrosomal Cep152 [42]. From the structural point of view, it would be interesting to determine the high-resolution structures of the C-terminal *β*-strand-rich region in SPD-2 and investigate how they are involved in centriole duplication (figure 2*b*). To determine the high-resolution structures of Asterless and Cep152 might prove challenging because of their high coiled-coil content (figure 2*c*). Reconstitution of a complex of the coiled-coil with an interaction partner might help to make its structural studies amenable. The MitoCheck consortium has reported that at least nine other proteins interact with Cep152 in mammalian cells [43].

## 3. Plk4/ZYG-1

**Figure 3.**
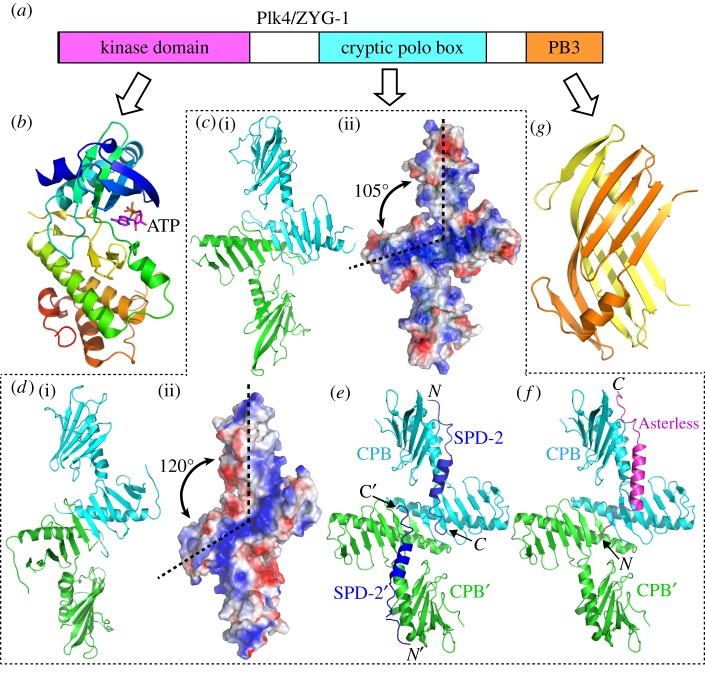
Structural dissection of Plk4/ZYG-1. (*a*) Schematic showing domain organization of Plk4/ZYG-1. (*b*) Ribbon diagram of the crystal structure of the human Plk4 kinase domain loaded with an ATP analogue (PDB code: 3COK). The structure is colour-ramped from blue (N-terminus) to red (C-terminus). The bound nucleotide is shown as sticks. (*c*) (i) Ribbon diagram and (ii) corresponding electrostatic surface plot of the crystal structure of the *Drosophila* Plk4 CPB dimer (PDB code: 4NK7). (*d*) (i) Ribbon diagram and (ii) corresponding electrostatic surface plot of the crystal structure of the ZYG-1 CPB dimer (PDB code: 4NKB). (*e*) Crystal structure of human Plk4 CPB in complex with the acidic region of SPD-2 (dark blue) (PDB code: 4N7Z). (*f*) Crystal structure of human Plk4 CPB in complex with the acidic region of Asterless (pink) (PDB code: 4N7 V). (*g*) Ribbon diagram of the crystal structure of the mouse Plk4 PB3 (PDB code: 1MBY). Different molecules in all ribbon diagrams are coloured differently. Positive and negative charges in the electrostatic plots are shown in blue and red, respectively.

In metazoans, the serine/threonine kinase Plk4 controls daughter centriole assembly and couples centriole duplication with cell-cycle progression [44,45]. As the master regulators of centriole assembly, Plk4 and ZYG-1 are essential for the recruitment of downstream proteins to the procentriole [24,25,46]. In *C. elegans*, ZYG-1 directly binds SAS-6 in order to recruit it and its interaction partner SAS-5 to the mother centriole [47]. In *Drosophila* and mammals, Plk4 phosphorylates Ana2/STIL to trigger the direct interaction between Ana2/STIL and SAS-6, which is essential for their recruitment during centriole assembly [48–51]. Plk4 is the most divergent member of the polo-like kinase family, and *C. elegans* ZYG-1 is one of the most divergent of Plk4 orthologues [52]. Despite the high divergence in their primary sequences, ZYG-1 and Plk4 share a similar structural arrangement consisting of three structural domains: the N-terminal kinase domain, the central CPB domain and the C-terminal single polo-box (PB3) domain (figure 3*a*).

The kinase domain of Plk4 adopts a similar conformation to that of Plk1, with the nucleotide binding site located in the cleft between two lobe-like modules (figure 3*b*). The central and C-terminal regions of Plk4 and ZYG-1 are the least similar in terms of protein sequence comparison. It has been debated whether ZYG-1 is a *bona fide* Plk4 or a structurally distinct functional orthologue [44]. To address this, it was critical to compare their respective CPB and PB3 domains to find out how similar they really are in three-dimensional structure. Initially, a crystal structure of the *Drosophila* Plk4 CPB was determined, based on which a side-by-side dimeric conformation was proposed [53]. However, this orientation of the Plk4 CPBs was later found to be a crystallographic artefact [26,54]. Other more recent structural studies on the ZYG-1 and Plk4 CPBs revealed that they in fact form a similar Z-shaped dimer, with a basic patch across the dimeric interface for binding by SPD-2 and/or Asterless [26,55]. Interestingly, despite the high similarity in their overall conformation, there are subtle structural variations in the CPBs of ZYG-1 and Plk4 that confer their selective binding to the respective receptors [26]. The CPB of *Drosophila* Plk4 bears a flat and broad basic patch (figure 3*c*), whereas the basic patch on the ZYG-1 CPB is narrow and elongated because of the expanded angle between the arms (figure 3*d*). A crystal structure of the human Plk4 CPB revealed that it is very similar to that of the *Drosophila* Plk4 CPB [55]. The structure of the human Plk4 CPB in complex with either SPD-2 (figure 3*e*) or Asterless (figure 3*f*) showed that the two receptors are bound with distinct modes, suggesting an intricate spatio-temporal regulation of Plk4 recruitment [55].

A crystal structure of the C-terminal PB3 of mouse Plk4 showed that it forms a dimeric structure distinct from the canonical polo-box (figure 3*g*) [56]. Recent studies suggested that the PB3 of human Plk4 plays an important regulatory role by relieving auto-inhibition of the kinase domain [57]. Similarly, the C-terminal PB3 in ZYG-1 has also been shown to play a regulatory role in its function. ZYG-1 deletion mutations lacking its PB3 make the kinase behave differently in mitosis and meiosis [58]. It was proposed that the ZYG-1 C-terminus may possess two distinct regulatory roles: one for inhibiting the kinase activity and the other for differential regulation of ZYG-1 recruitment in mitosis and meiosis [58]. However, the underlying mechanism for how the PB3 regulates ZYG-1 activity remains elusive. A crystal structure of the mouse Plk4 PB3 dimer revealed a deep interfacial pocket between the two molecules, which was proposed to be a potential ligand-binding site [56]. However, no ligands binding to this pocket of the Plk4 PB3 have yet been identified. Recent studies further demonstrated that the human Plk4 PB3 does not dimerize [57]. Therefore, it remains unclear whether the PB3 of Plk4 in different organisms indeed all forms a dimer and, if so, whether the ZYG-1 PB3 shares a similar conformation to it. Determination of high-resolution structures for the PB3 domains of ZYG-1 and various Plk4s would also help investigate how PB3 mechanistically regulates the function of Plk4 and ZYG-1 *in vivo*.

## 4. SAS-5/Ana2/STIL and SAS-6

SAS-5 and SAS-6 are co-dependent for their centriolar localization, which is essential for the subsequent recruitment of SAS-4 [23]. SAS-5 is functionally orthologous to *Drosophila* Ana2 and vertebrate STIL, with a 90-residue STIL/Ana2 (STAN) motif at the C-terminus [59]. Reflecting evolutionary history, this motif is more conserved among vertebrates and flies (greater than 30% identity) than with the corresponding region in *C. elegans* SAS-5 (12% identity). Likewise, the binding modes of Ana2 and SAS-5 to their respective partners seem to be different. While the binding of SAS-5 to SAS-6 is mediated via synergistic hydrophobic and electrostatic interactions between the C-terminal 15 residues of SAS-5 (which is predicted to form a short *α*-helix) and a central region of the SAS-6 coiled-coil [60], such an interaction seems to be absent in *Drosophila* and mammals. This is despite the fact that both Ana2 and STIL are also predicted to possess a short helix towards their C-termini and that two of the four residues at the C-terminus of SAS-5 for SAS-6 binding are also conserved in this helix of Ana2 and STIL. Instead, the interaction between Ana2/STIL and SAS-6 was recently revealed to be mediated by the Plk4-dependent phosphorylation of a number of conserved serine/threonine residues in the STAN motif of Ana2/STIL [48–51]. ZYG-1 was initially reported to phosphorylate SAS-6 at serine 123 residue [61]. However, later studies with mutations of 38 potential phosphorylation sites in *C. elegans* SAS-6 (including serine123) suggest that SAS-6 is unlikely to be the phosphorylation target of ZYG-1 [47]. It remains to be determined whether ZYG-1 phosphorylates SAS-5 in a similar manner to that of Plk4 on Ana2/STIL.

**Figure 4.**
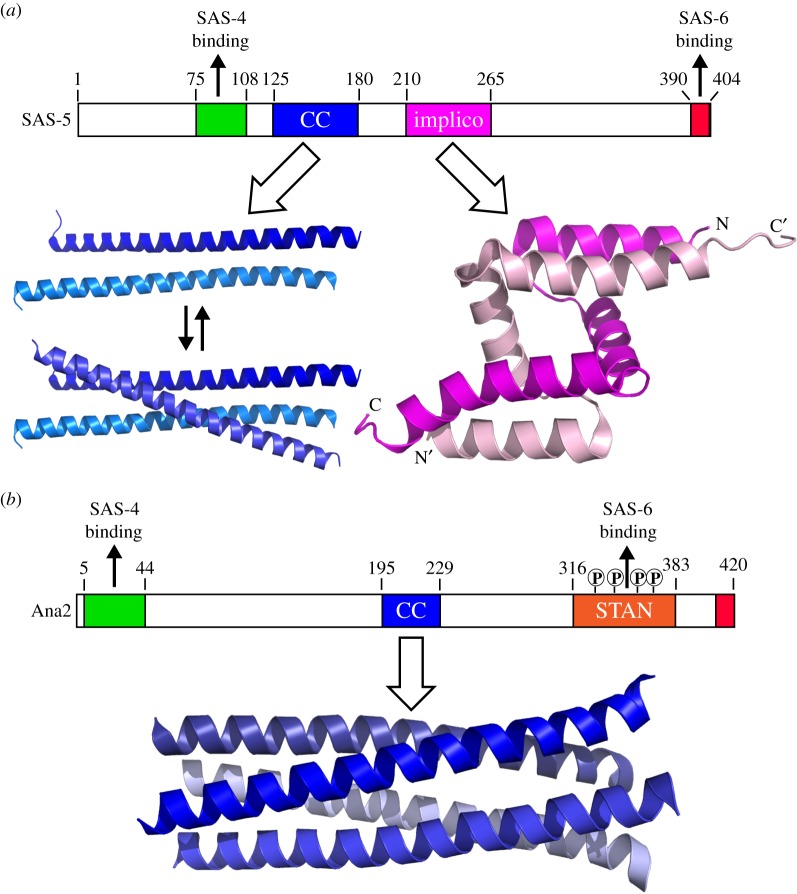
Domain arrangements and crystal structures of SAS-5 and Ana2. (*a*) Domain organization and crystal structures of SAS-5. The central coiled-coil may form a dimer or trimer depending on the protein concentration. The *Implico* domain forms an intertwined homodimer. The C-terminal 15 residues of SAS-5 (shown in red) are predicted to form an *α*-helix that binds directly to a central region of the SAS-6 coiled-coil. (*b*) Domain organization of *Drosophila* Ana2 and crystal structure of its parallel, symmetrical 4-helix bundle. Plk4 phosphorylates four conserved serine residues in the STAN motif to promote the strong interaction between Ana2 and SAS-6.

Studies using various biophysical methods suggested that the central region of SAS-5 forms a tetramer, which may function as a cross-linker to strengthen the higher-order structure formed by SAS-6 [62]. Detailed molecular mechanisms underlying SAS-5 oligomerization have been revealed very recently. Crystal structures show that SAS-5 contains two independent oligomeric domains: a coiled-coil region spanning residues 125–180 and an *Implico* domain consisting of residues 210–265 (figure 4*a*) [63]. While the *Implico* motif forms a stable dimer via the intertwining of the zigzagged triple-helix structures, the coiled-coil can exist as either a dimer or trimer depending on its concentration. Together, these properties allow SAS-5 to form a higher-order assembly that may create a seeding point for SAS-6 oligomerization [63]. In the meantime, a crystal structure of the central coiled-coil domain of Ana2 has been reported, which reveals that it forms a parallel, symmetrical 4-helix bundle (figure 4*b*) [64]. This tetrameric form, together with its N-terminal SAS-4 binding site and C-terminal phosporylation-dependent SAS-6-binding STAN motif, was believed to actively contribute to centriole assembly by interconnecting neighbouring layers of cartwheels formed by SAS-6 [64]. While auto-assembly of both SAS-5 and Ana2 is essential for centriole biogenesis, their different oligomerization modes might reflect the morphological variations of the respective SAS-6 oligomers that have been observed (see below).

**Figure 5.**
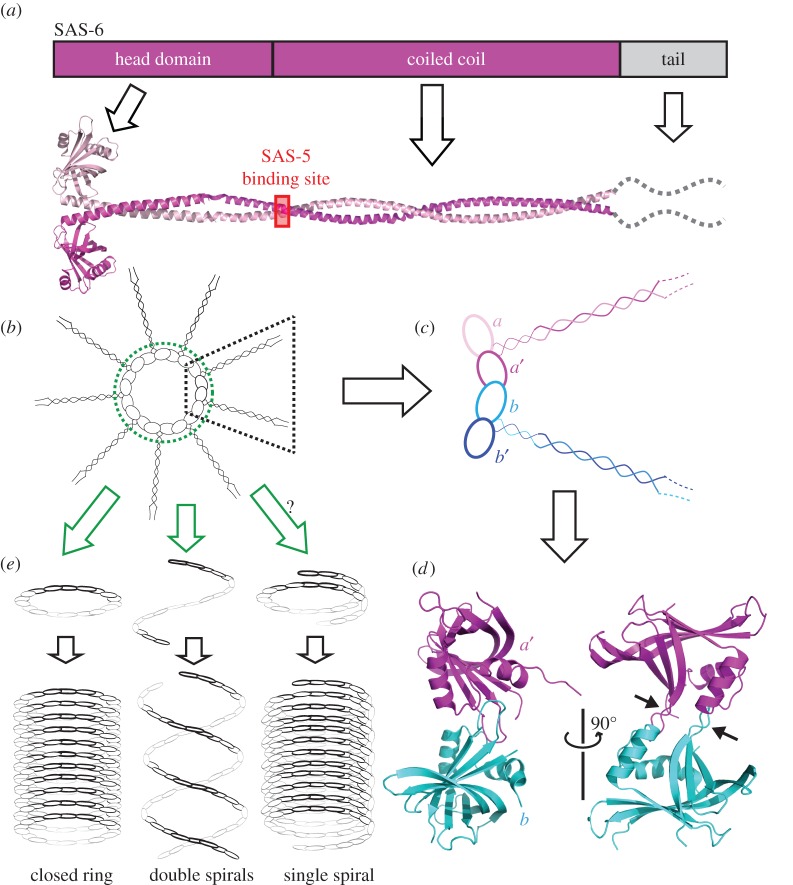
Structure and assembly of SAS-6. (*a*) Domain organization and ribbon diagram representation of SAS-6. SAS-6 molecules form a parallel dimer via their coiled-coil domains. The SAS-5 binding site is indicated. (*b*) Assembly of nine SAS-6 dimers into a cartwheel-like structure via intermolecular interactions of the head domains around the ring. (*c*) Coloured schematic showing the head–head interaction between two neighbouring SAS-6 dimers. (*d*) Ribbon diagram of the crystal structure of the *C. elegans* SAS-6 head domain dimer (PDB code: 3PYI). Arrows point to the loops mediating the intermolecular interaction. (*e*) Three working models for formation of the cylinder-like structure by SAS-6. The spokes formed by SAS-6 coiled-coils are omitted for clarity.

SAS-6 is one of the most thoroughly characterized centriolar proteins at a structural level. It is a modular protein with an N-terminal head domain followed by a long coiled-coil and an intrinsically disordered C-terminal tail (figure 5*a*). Numerous crystal structures of the head domain of SAS-6 from various organisms have been reported, which show a conserved intermolecular interaction between the head domains [65–68]. Furthermore, a crystal structure of the *C. elegans* SAS-6 coiled-coil region shows that two SAS-6 molecules form a parallel dimer via a strong interaction between their coiled-coils [60]. Together, these studies suggest that 18 SAS-6 molecules can form nine parallel homodimers that then assemble into a cartwheel-like structure. The cartwheel is stabilized through the interactions between the neighbouring head domains in the closed ring (figure 5*b–d*). This working model finally provides a molecular explanation for the ninefold cartwheel structure originally identified by electron microscopy [69].

While the cartwheel formed by SAS-6 is believed to be important for dictating the ninefold symmetry of centrioles, the interaction between the SAS-6 head domains is relatively weak, with a *K*_d_ of only 60–100 µM. This argues against the autonomous assembly of the cartwheel by SAS-6 alone. In the cell, this issue might be solved via the cross-linking action of SAS-5/Ana2/STIL as described above. Interestingly, it was shown recently that in human cells cartwheel assembly occurs in the proximal lumen of the mother centriole [70]. This assembly process is initiated by the recruitment of SAS-6 dimers to disengaged centrioles, and mediated by the interaction between the SAS-6 C-terminal tail and SAS-4 located at the luminal wall [70]. However, this seems to be species-specific and might be employed only by cycling cells with strictly controlled centriole number, as *de novo* assembly of centrioles has been observed in many other species [71–73].

To form a cylindrical centriole, the SAS-6-mediated cartwheel structure apparently has to be assembled longitudinally in the lumen of the centriole. One way to achieve this is by stacking multiple rings on top of one another, as observed in the flagellate *Trichonympha* (figure 5*e*, left) [74]. This assembly mechanism might also occur in other organisms [75,76]. The other means of SAS-6 assembly was found in *C. elegans* where SAS-6 forms a spiral-like structure, and two such spirals wind around each other to form the central tube of the centriole (figure 5*e*, middle) [65]. It is postulated that SAS-6 might also assemble in a third way, in which SAS-6 forms a single tight spiral formed by the slightly tilted hinge region between the head group and the coiled-coil (figure 5*e*, right) [77]. However, the single spiral model has not been seen in any real centrioles so far and its existence remains undetermined.

## 5. SAS-4/CPAP

**Figure 6.**
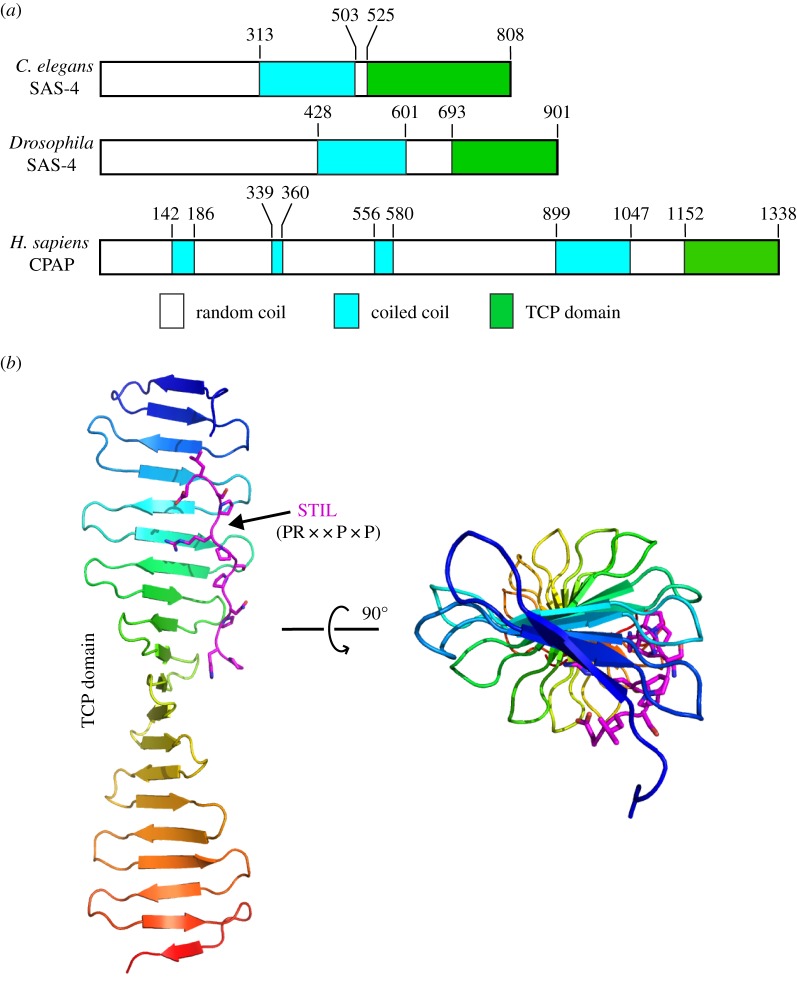
Domain organization and structural dissection of SAS-4/CPAP. (*a*) Domain organization of three representative SAS-4/CPAP homologues. All SAS-4/CPAP homologues share a similar structural arrangement with a mostly disordered N-terminus, a central coiled-coil and a β-strand-rich C-terminal TCP domain. Residue numbers of domain boundaries are shown above the schematics. (*b*) Ribbon diagram of the crystal structure of the TCP domain of *Danio rerio* CPAP in complex with the ‘PR××P×P’ motif from STIL. The motif is shown as sticks (PDB code: 4BXR). The complex is shown in two perpendicular orientations.

SAS-4/CPAP is the last core centriolar protein to be recruited. It is located at the outer wall of the centriolar barrel and plays an essential role for subsequent recruitment of microtubules [24,25]. SAS-4/CPAP was originally identified in mammals by yeast two-hybrid screens [78]. Homologues of SAS-4/CPAP were also later identified in *Drosophila* and *C. elegans* [19,20,79]. All SAS-4/CPAP proteins share a similar overall domain arrangement, with a mostly disordered N-terminus, a central coiled-coil and a globular C-terminal T complex protein 10 (TCP) domain (figure 6*a*). Crystal structures of the TCP domain of SAS-4 proteins from zebrafish and *Drosophila* showed that it forms a long β-meander consisting of about 20 β-strands, which interacts directly with a conserved proline-rich region in STIL/Ana2—the consensus sequence of this region is PR××P×P (figure 6*b*) [80,81]. Interestingly, the seemingly stable TCP structure does not contain a defined hydrophobic core or flanking globular domains on either end.

While the interaction mode between the SAS-4 TCP domain and STIL/Ana2 is conserved in *Drosophila* and vertebrates, the interaction between *C. elegans* SAS-4 and SAS-5 may have diverged significantly. For instance, although the TCP domain is required for *C. elegans* SAS-4 to interact with the N-terminal part of SAS-5, by itself it is not sufficient [80]. Furthermore, SAS-5 does not contain the consensus TCP-binding motif (PR××P×P) present in STIL/Ana2 [80].

The function of SAS-4/CPAP in recruiting microtubules during centriole assembly has been confirmed. However, the mechanism is still unclear. One hypothesis is that the TCP domain of *Drosophila* CPAP forms monomers in solution, but upon incorporation into procentrioles the TCP domain would bind to STIL towards the interior of the centriole whereas the N-terminal part of SAS-4/CPAP interacts with Asterless/Cep152 and microtubules at the outer wall [80]. Another is that the TCP β-sheet is capable of forming large assemblies via a head-to-tail interaction. This was observed in the crystal structure and was postulated to help CPAP to form continuous connections along the long axis of the centriolar outer wall. Such connections, together with the central coiled-coil of the protein, would then link the pinhead with the microtubules to stabilize the cartwheel stacks in the centriole [81]. Structural and functional analysis of *Drosophila* SAS-4 TCP domain by another group further confirmed the role of SAS-4 as a mediator to tether PCM complexes to the centriole to facilitate centrosome assembly [82]. However, further studies are needed to find out the exact structural roles of SAS-4/CPAP in centriole assembly.

## 6. Other proteins involved in centriole assembly

Besides the five core centriolar components, a number of other proteins participating in centriole duplication have recently been identified. Bld10/Cep135 is an approximately 170 kDa protein originally found in *Chlamydomonas* and essential for cartwheel formation [83]. It consists mostly of coiled-coils and was later found to be a major spoke-tip component, serving to bridge the cartwheel to the triplet microtubules [84]. A homologue of Bld10/Cep135 was subsequently identified in *Drosophila*, and was reported to play a role in the formation of both the central tube in mature primary spermatocytes [85] and the central pair of microtubules in the flagellar axoneme [86,87]. However, no Bld10/Cep135 homologue has been identified in nematodes.

The localization of Bld10/Cep135 to procentrioles depends on both Cep120 and SPICE1 [88]. Cep120 is an approximately 100 kDa protein participating in centriole assembly by asymmetrically localizing to the daughter centriole [89]. The protein contains an N-terminal C2 domain, a central region for SAS-4/CPAP binding and a C-terminal long coiled-coil for dimerization [90]. It was reported that Cep120 interacts with SPICE1, another large coiled-coil-containing protein, and the two proteins are co-dependent for their localization to procentrioles [88]. Cep120 and SPICE1 are believed to work cooperatively with SAS-4/CPAP to regulate centriole elongation [88,90].

Comparative genomics and proteomics studies of isolated basal bodies from *Chlamydomonas reinhardtii* have identified 18 proteins (Poc 1–Poc 18) proposed to be centriolar constituents [91]. Poc1 is one of the best-characterized Poc proteins. It contains an N-terminal WD40 domain and a C-terminal coiled-coil and, like Bld10/Cep135, is hypothesized to stabilize microtubules, and thus maintains centriole assembly and integrity [92–95].

In animal cells, a cap structure at the distal end of centrioles is formed to suppress centriole-templated ciliogenesis. The cap complex consists of CP110 and Cep97, two large proteins consisting mostly of coiled-coils [96]. Additionally, several other proteins have been identified to be more specifically involved in assembly of an appendage structure at the distal end of mother centrioles. These proteins include Ccdc41/Cep83, Cep89/Cep123, Sclt1, Fbf1 and Cep164 [97–99]. The distal appendage serves to anchor the mother centriole onto the plasma membrane during ciliogenesis.

## 7. Conclusion and perspectives

The recent progress in structural studies on centriolar components, with each of the five core components having been at least partially characterized, has greatly advanced our understanding of centriole assembly at the high-resolution level. However, further studies will be necessary to precisely determine their contributions, both physically and temporally, during centriole assembly, and to uncover their interplay with other newly identified constitutive or accessory centriolar proteins and each other. The field faces several challenges in this process including (i) to understand how centriole assembly is initiated, (ii) to uncover how centriole duplication is regulated by both kinases and phosphatases, (iii) to determine the high-resolution structures and the functional roles for those coiled-coil-containing centriolar proteins, and (iv) to precisely assign the relative positions and the interaction network of different centriolar proteins and their structural/functional roles.

Recent studies using various techniques have identified many new centriolar components. It is likely that more centriole-associated proteins will be reported in the future. However, unlike the five core centriolar proteins, at least some of them are organism- or cell-specific. For example, although Bld10/Cep135 is crucial for cartwheel formation in green algae [83,84], *Paramecium tetraurelia* [100] and mammalian cells [46,101], its homologue in *Drosophila* is dispensable for cartwheel formation [85,87,102], and no homologue of Bld10/Cep135 seems to exist in nematodes. Another example is CP110 and Cep97, both of which exist only in animals, and are needed for centriole duplication in S-phase-arrested human cells to control centriole length [46,103–105]. Phylogenetic analysis suggests that both CP110 and Cep97 might have been recruited to the centriole-assembly pathway in animals during evolution to coordinate centriole assembly with ciliogenesis and/or cytokinesis [86]. Therefore, it is necessary to carefully examine which of these newly identified centriolar proteins are structural constituents of the centriole and which are accessory proteins, and what their structural and functional roles are in centriole assembly.

It is foreseeable that many more structural characterizations on centriole assembly will be reported in the coming years, which, together with continuous advancements in functional and *in vivo* studies, will provide a more refined view of one of the most important and fascinating cellular structures that has captivated cell biologists for over a century.

## Note added in proof

Arquint *et al*. [106] just reported high-resolution structures of the PB3 of human Plk4, both alone and in complex with STIL. Their studies demonstrate that the human plk4 PB3, unlike the previously reported dimeric mouse Plk4 PB3 (PDB code: 1MBY), folds into a monomeric canonical polo-box and binds to the central coiled-coil of STIL via a novel polo-box—peptide interaction mode mimicking coiled-coil formation.

## References

[1] Marshall WF 2001 Centrioles take center stage. Curr. Biol. 11, R487–R496. (doi:10.1016/S0960-9822(01)00289-5) 11448793 10.1016/s0960-9822(01)00289-5

[2] Preble AM, Giddings TM Jr, Dutcher SK 2000 Basal bodies and centrioles: their function and structure. Curr. Top. Dev. Biol. 49, 207–233. (doi:10.1016/S0070-2153(99)49010-6) 11005020 10.1016/s0070-2153(99)49010-6

[3] Bobinnec Y, Khodjakov A, Mir LM, Rieder CL, Edde B, Bornens M 1998 Centriole disassembly *in vivo* and its effect on centrosome structure and function in vertebrate cells. J. Cell Biol. 143, 1575–1589. (doi:10.1083/jcb.143.6.1575) 9852152 10.1083/jcb.143.6.1575 PMC2132987

[4] Bornens M 2002 Centrosome composition and microtubule anchoring mechanisms. Curr. Opin. Cell Biol. 14, 25–34. (doi:10.1016/S0955-0674(01)00290-3) 11792541 10.1016/s0955-0674(01)00290-3

[5] Conduit PT et al. 2014 The centrosome-specific phosphorylation of Cnn by Polo/Plk1 drives Cnn scaffold assembly and centrosome maturation. Dev. Cell 28, 659–669. (doi:10.1016/j.devcel.2014.02.013) 10.1016/j.devcel.2014.02.013 PMC3988887 24656740

[6] Conduit PT et al. 2014 A molecular mechanism of mitotic centrosome assembly in *Drosophila*. eLife 3, e03399 (doi:10.7554/eLife.03399) 25149451 10.7554/eLife.03399 PMC4175739

[7] Mennella V, Agard DA, Huang B, Pelletier L 2014 Amorphous no more: subdiffraction view of the pericentriolar material architecture. Trends Cell Biol. 24, 188–197. (doi:10.1016/j.tcb.2013.10.001) 10.1016/j.tcb.2013.10.001 PMC3991556 24268653

[8] Woodruff JB, Wueseke O, Hyman AA 2014 Pericentriolar material structure and dynamics. Phil. Trans. R. Soc. B 369, 20130459 (doi:10.1098/rstb.2013.0459) 25047613 10.1098/rstb.2013.0459 PMC4113103

[9] Anderhub SJ, Kramer A, Maier B 2012 Centrosome amplification in tumorigenesis. Cancer Lett. 322, 8–17. (doi:10.1016/j.canlet.2012.02.006) 22342684 10.1016/j.canlet.2012.02.006

[10] Bettencourt-Dias M, Hildebrandt F, Pellman D, Woods G, Godinho SA 2011 Centrosomes and cilia in human disease. Trends Genet. 27, 307–315. (doi:10.1016/j.tig.2011.05.004) 21680046 10.1016/j.tig.2011.05.004 PMC3144269

[11] Brinkley BR 2001 Managing the centrosome numbers game: from chaos to stability in cancer cell division. Trends Cell Biol. 11, 18–21. (doi:10.1016/S0962-8924(00)01872-9) 11146294 10.1016/s0962-8924(00)01872-9

[12] Nigg EA 2006 Origins and consequences of centrosome aberrations in human cancers. Int. J. Cancer 119, 2717–2723. (doi:10.1002/ijc.22245) 17016823 10.1002/ijc.22245

[13] Satir P, Christensen ST 2007 Overview of structure and function of mammalian cilia. Annu. Rev. Physiol. 69, 377–400. (doi:10.1146/annurev.physiol.69.040705.141236) 17009929 10.1146/annurev.physiol.69.040705.141236

[14] Adams M, Smith UM, Logan CV, Johnson CA 2008 Recent advances in the molecular pathology, cell biology and genetics of ciliopathies. J. Med. Genet. 45, 257–267. (doi:10.1136/jmg.2007.054999) 18178628 10.1136/jmg.2007.054999

[15] Mahjoub MR 2013 The importance of a single primary cilium. Organogenesis 9, 61–69. (doi:10.4161/org.25144) 23819944 10.4161/org.25144 PMC3812286

[16] O'Connell KF, Caron C, Kopish KR, Hurd DD, Kemphues KJ, Li Y, White JG 2001 The *C. elegans* zyg-1 gene encodes a regulator of centrosome duplication with distinct maternal and paternal roles in the embryo. Cell 105, 547–558. (doi:10.1016/S0092-8674(01)00338-5) 11371350 10.1016/s0092-8674(01)00338-5

[17] Kemp CA, Kopish KR, Zipperlen P, Ahringer J, O'Connell KF 2004 Centrosome maturation and duplication in *C. elegans* require the coiled-coil protein SPD-2. Dev. Cell 6, 511–523. (doi:10.1016/S1534-5807(04)00066-8) 15068791 10.1016/s1534-5807(04)00066-8

[18] Pelletier L, Ozlu N, Hannak E, Cowan C, Habermann B, Ruer M, Muller-Reichert T, Hyman AA 2004 The *Caenorhabditis elegans* centrosomal protein SPD-2 is required for both pericentriolar material recruitment and centriole duplication. Curr. Biol. 14, 863–873. (doi:10.1016/j.cub.2004.04.012) 15186742 10.1016/j.cub.2004.04.012

[19] Kirkham M, Muller-Reichert T, Oegema K, Grill S, Hyman AA 2003 SAS-4 is a *C. elegans* centriolar protein that controls centrosome size. Cell 112, 575–587. (doi:10.1016/S0092-8674(03)00117-X) 12600319 10.1016/s0092-8674(03)00117-x

[20] Leidel S, Gonczy P 2003 SAS-4 is essential for centrosome duplication in *C. elegans* and is recruited to daughter centrioles once per cell cycle. Dev. Cell 4, 431–439. (doi:10.1016/S1534-5807(03)00062-5) 12636923 10.1016/s1534-5807(03)00062-5

[21] Delattre M, Leidel S, Wani K, Baumer K, Bamat J, Schnabel H, Feichtinger R, Schnabel R, Gonczy P 2004 Centriolar SAS-5 is required for centrosome duplication in *C. elegans*. Nat. Cell Biol. 6, 656–664. (doi:10.1038/ncb1146) 15232593 10.1038/ncb1146

[22] Dammermann A, Muller-Reichert T, Pelletier L, Habermann B, Desai A, Oegema K 2004 Centriole assembly requires both centriolar and pericentriolar material proteins. Dev. Cell 7, 815–829. (doi:10.1016/j.devcel.2004.10.015) 15572125 10.1016/j.devcel.2004.10.015

[23] Leidel S, Delattre M, Cerutti L, Baumer K, Gonczy P 2005 SAS-6 defines a protein family required for centrosome duplication in *C. elegans* and in human cells. Nat. Cell Biol. 7, 115–125. (doi:10.1038/ncb1220) 15665853 10.1038/ncb1220

[24] Delattre M, Canard C, Gonczy P 2006 Sequential protein recruitment in *C. elegans* centriole formation. Curr. Biol. 16, 1844–1849. (doi:10.1016/j.cub.2006.07.059) 16979563 10.1016/j.cub.2006.07.059

[25] Pelletier L, O'Toole E, Schwager A, Hyman AA, Muller-Reichert T 2006 Centriole assembly in *Caenorhabditis elegans*. Nature 444, 619–623. (doi:10.1038/nature05318) 17136092 10.1038/nature05318

[26] Shimanovskaya E, Viscardi V, Lesigang J, Lettman MM, Qiao R, Svergun DI, Round A, Oegema K, Dong G 2014 Structure of the *C. elegans* ZYG-1 cryptic polo box suggests a conserved mechanism for centriolar docking of Plk4 kinases. Structure 22, 1090–1104. (doi:10.1016/j.str.2014.05.009) 24980795 10.1016/j.str.2014.05.009 PMC4126857

[27] Dix CI, Raff JW 2007 Drosophila Spd-2 recruits PCM to the sperm centriole, but is dispensable for centriole duplication. Curr. Biol. 17, 1759–1764. (doi:10.1016/j.cub.2007.08.065) 17919907 10.1016/j.cub.2007.08.065 PMC2045633

[28] Giansanti MG, Bucciarelli E, Bonaccorsi S, Gatti M 2008 Drosophila SPD-2 is an essential centriole component required for PCM recruitment and astral-microtubule nucleation. Curr. Biol. 18, 303–309. (doi:10.1016/j.cub.2008.01.058) 18291647 10.1016/j.cub.2008.01.058

[29] Bonaccorsi S, Giansanti MG, Gatti M 1998 Spindle self-organization and cytokinesis during male meiosis in asterless mutants of *Drosophila melanogaster*. J. Cell Biol. 142, 751–761. (doi:10.1083/jcb.142.3.751) 9700163 10.1083/jcb.142.3.751 PMC2148166

[30] Blachon S, Gopalakrishnan J, Omori Y, Polyanovsky A, Church A, Nicastro D, Malicki J, Avidor-Reiss T 2008 *Drosophila* Asterless and vertebrate Cep152 are orthologs essential for centriole duplication. Genetics 180, 2081–2094. (doi:10.1534/genetics.108.095141) 18854586 10.1534/genetics.108.095141 PMC2600943

[31] Dobbelaere J, Josue F, Suijkerbuijk S, Baum B, Tapon N, Raff J 2008 A genome-wide RNAi screen to dissect centriole duplication and centrosome maturation in *Drosophila*. PLoS Biol. 6, e224 (doi:10.1371/journal.pbio.0060224) 18798690 10.1371/journal.pbio.0060224 PMC2535660

[32] Dzhindzhev NS et al. 2010 Asterless is a scaffold for the onset of centriole assembly. Nature 467, 714–718. (doi:10.1038/nature09445) 20852615 10.1038/nature09445

[33] Varmark H, Llamazares S, Rebollo E, Lange B, Reina J, Schwarz H, Gonzalez C 2007 Asterless is a centriolar protein required for centrosome function and embryo development in *Drosophila*. Curr. Biol. 17, 1735–1745. (doi:10.1016/j.cub.2007.09.031) 17935995 10.1016/j.cub.2007.09.031

[34] Cizmecioglu O, Arnold M, Bahtz R, Settele F, Ehret L, Haselmann-Weiss U, Antony C, Hoffmann I 2010 Cep152 acts as a scaffold for recruitment of Plk4 and CPAP to the centrosome. J. Cell Biol. 191, 731–739. (doi:10.1083/jcb.201007107) 21059844 10.1083/jcb.201007107 PMC2983070

[35] Firat-Karalar EN, Rauniyar N, Yates JR 3rd, Stearns T 2014 Proximity interactions among centrosome components identify regulators of centriole duplication. Curr. Biol. 24, 664–670. (doi:10.1016/j.cub.2014.01.067) 24613305 10.1016/j.cub.2014.01.067 PMC4004176

[36] Hatch EM, Kulukian A, Holland AJ, Cleveland DW, Stearns T 2010 Cep152 interacts with Plk4 and is required for centriole duplication. J. Cell Biol. 191, 721–729. (doi:10.1083/jcb.201006049) 21059850 10.1083/jcb.201006049 PMC2983069

[37] Klebba JE, Galletta BJ, Nye J, Plevock KM, Buster DW, Hollingsworth NA, Slep KC, Rusan NM, Rogers GC 2015 Two Polo-like kinase 4 binding domains in Asterless perform distinct roles in regulating kinase stability. J. Cell Biol. 208, 401–414. (doi:10.1083/jcb.201410105) 25688134 10.1083/jcb.201410105 PMC4332252

[38] Wueseke O et al. 2014 The *Caenorhabditis elegans* pericentriolar material components SPD-2 and SPD-5 are monomeric in the cytoplasm before incorporation into the PCM matrix. Mol. Biol. Cell 25, 2984–2992. (doi:10.1091/mbc.E13-09-0514) 25103243 10.1091/mbc.E13-09-0514 PMC4230587

[39] Loncarek J, Hergert P, Khodjakov A 2010 Centriole reduplication during prolonged interphase requires procentriole maturation governed by Plk1. Curr. Biol. 20, 1277–1282. (doi:10.1016/j.cub.2010.05.050) 20656208 10.1016/j.cub.2010.05.050 PMC2911792

[40] Tsou MF, Stearns T 2006 Mechanism limiting centrosome duplication to once per cell cycle. Nature 442, 947–951. (doi:10.1038/nature04985) 16862117 10.1038/nature04985

[41] Wang WJ, Soni RK, Uryu K, Tsou MF 2011 The conversion of centrioles to centrosomes: essential coupling of duplication with segregation. J. Cell Biol. 193, 727–739. (doi:10.1083/jcb.201101109) 21576395 10.1083/jcb.201101109 PMC3166877

[42] Sir JH et al. 2011 A primary microcephaly protein complex forms a ring around parental centrioles. Nat. Genet. 43, 1147–1153. (doi:10.1038/ng.971) 21983783 10.1038/ng.971 PMC3299569

[43] Hutchins JR et al. 2010 Systematic analysis of human protein complexes identifies chromosome segregation proteins. Science 328, 593–599. (doi:10.1126/science.1181348) 20360068 10.1126/science.1181348 PMC2989461

[44] Cunha-Ferreira I et al. 2013 Regulation of autophosphorylation controls PLK4 self-destruction and centriole number. Curr. Biol. 23, 2245–2254. (doi:10.1016/j.cub.2013.09.037) 24184099 10.1016/j.cub.2013.09.037

[45] Sillibourne JE, Bornens M 2010 Polo-like kinase 4: the odd one out of the family. Cell Div. 5, 25 (doi:10.1186/1747-1028-5-25) 20920249 10.1186/1747-1028-5-25 PMC2955731

[46] Kleylein-Sohn J, Westendorf J, Le Clech M, Habedanck R, Stierhof YD, Nigg EA 2007 Plk4-induced centriole biogenesis in human cells. Dev. Cell 13, 190–202. (doi:10.1016/j.devcel.2007.07.002) 17681131 10.1016/j.devcel.2007.07.002

[47] Lettman MM, Wong YL, Viscardi V, Niessen S, Chen SH, Shiau AK, Zhou HL, Desai A, Oegema K 2013 Direct binding of SAS-6 to ZYG-1 recruits SAS-6 to the mother centriole for cartwheel assembly. Dev. Cell 25, 284–298. (doi:10.1016/j.devcel.2013.03.011) 23673331 10.1016/j.devcel.2013.03.011 PMC3655416

[48] Dzhindzhev NS, Tzolovsky G, Lipinszki Z, Schneider S, Lattao R, Fu J, Debski J, Dadlez M, Glover DM 2014 Plk4 phosphorylates Ana2 to trigger Sas6 recruitment and procentriole formation. Curr. Biol. 24, 2526–2532. (doi:10.1016/j.cub.2014.08.061) 25264260 10.1016/j.cub.2014.08.061 PMC4229625

[49] Kratz AS, Barenz F, Richter KT, Hoffmann I 2015 Plk4-dependent phosphorylation of STIL is required for centriole duplication. Biol. Open 4, 370–377. (doi:10.1242/bio.201411023) 25701666 10.1242/bio.201411023 PMC4359743

[50] Moyer TC, Clutario KM, Lambrus BG, Daggubati V, Holland AJ 2015 Binding of STIL to Plk4 activates kinase activity to promote centriole assembly. J. Cell Biol. 209, 863–878. (doi:10.1083/jcb.201502088) 26101219 10.1083/jcb.201502088 PMC4477857

[51] Ohta M, Ashikawa T, Nozaki Y, Kozuka-Hata H, Goto H, Inagaki M, Oyama M, Kitagawa D 2014 Direct interaction of Plk4 with STIL ensures formation of a single procentriole per parental centriole. Nat. Commun. 5, 5267 (doi:10.1038/ncomms6267) 25342035 10.1038/ncomms6267 PMC4220463

[52] Jana SC, Bazan JF, Bettencourt-Dias M 2012 Polo boxes come out of the crypt: a new view of PLK function and evolution. Strucute 20, 1801–1804. (doi:10.1016/j.str.2012.10.008) 10.1016/j.str.2012.10.008 23141691

[53] Slevin LK, Nye J, Pinkerton DC, Buster DW, Rogers GC, Slep KC 2012 The structure of the plk4 cryptic polo box reveals two tandem polo boxes required for centriole duplication. Structure 20, 1905–1917. (doi:10.1016/j.str.2012.08.025) 23000383 10.1016/j.str.2012.08.025 PMC3496063

[54] Levine MS, Holland AJ 2014 Polo-like kinase 4 shapes up. Structure 22, 1071–1073. (doi:10.1016/j.str.2014.07.004) 25099950 10.1016/j.str.2014.07.004

[55] Park SY et al. 2014 Molecular basis for unidirectional scaffold switching of human Plk4 in centriole biogenesis. Nat. Struct. Mol. Biol. 21, 696–703. (doi:10.1038/nsmb.2846) 24997597 10.1038/nsmb.2846 PMC4125498

[56] Leung GC, Hudson JW, Kozarova A, Davidson A, Dennis JW, Sicheri F 2002 The Sak polo-box comprises a structural domain sufficient for mitotic subcellular localization. Nat. Struct. Biol. 9, 719–724. (doi:10.1038/nsb848) 12352953 10.1038/nsb848

[57] Klebba JE, Buster DW, McLamarrah TA, Rusan NM, Rogers GC 2015 Autoinhibition and relief mechanism for Polo-like kinase 4. Proc. Natl Acad. Sci. USA 112, E657–E666. (doi:10.1073/pnas.1417967112) 25646492 10.1073/pnas.1417967112 PMC4343128

[58] Peters N, Perez DE, Song MH, Liu Y, Muller-Reichert T, Caron C, Kemphues KJ, O'Connell KF 2010 Control of mitotic and meiotic centriole duplication by the Plk4-related kinase ZYG-1. J. Cell Sci. 123, 795–805. (doi:10.1242/jcs.050682) 20144993 10.1242/jcs.050682 PMC2823580

[59] Stevens NR, Roque H, Raff JW 2010 DSas-6 and Ana2 coassemble into tubules to promote centriole duplication and engagement. Dev. Cell 19, 913–919. (doi:10.1016/j.devcel.2010.11.010) 21145506 10.1016/j.devcel.2010.11.010 PMC4159445

[60] Qiao R, Cabral G, Lettman MM, Dammermann A, Dong G 2012 SAS-6 coiled-coil structure and interaction with SAS-5 suggest a regulatory mechanism in *C. elegans* centriole assembly. EMBO J. 31, 4334–4347. (doi:10.1038/emboj.2012.280) 23064147 10.1038/emboj.2012.280 PMC3501224

[61] Kitagawa D, Busso C, Fluckiger I, Gonczy P 2009 Phosphorylation of SAS-6 by ZYG-1 is critical for centriole formation in *C. elegans* embryos. Dev. Cell 17, 900–907. (doi:10.1016/j.devcel.2009.11.002) 20059959 10.1016/j.devcel.2009.11.002

[62] Shimanovskaya E, Qiao R, Lesigang J, Dong G 2013 The SAS-5 N-terminal domain is a tetramer, with implications for centriole assembly in *C. elegans*. Worm 2, e25214 (doi:10.4161/worm.25214) 24778935 10.4161/worm.25214 PMC3875647

[63] Rogala KB, Dynes NJ, Hatzopoulos GN, Yan J, Pong SK, Robinson CV, Deane CM, Gonczy P, Vakonakis I 2015 The *Caenorhabditis elegans* protein SAS-5 forms large oligomeric assemblies critical for centriole formation. eLife 4, e7410 (doi:10.7554/eLife.07410) 10.7554/eLife.07410 PMC4471805 26023830

[64] Cottee MA, Muschalik N, Johnson S, Leveson J, Raff JW, Lea SM 2015 The homo-oligomerisation of both Sas-6 and Ana2 is required for efficient centriole assembly in flies. eLife 4, e07236 (doi:10.7554/eLife.07236) 26002084 10.7554/eLife.07236 PMC4471874

[65] Hilbert M et al. 2013 *Caenorhabditis elegans* centriolar protein SAS-6 forms a spiral that is consistent with imparting a ninefold symmetry. Proc. Natl Acad. Sci. USA 110, 11 373–11 378. (doi:10.1073/pnas.1302721110) 10.1073/pnas.1302721110 PMC3710844 23798409

[66] Kitagawa D et al. 2011 Structural basis of the 9-fold symmetry of centrioles. Cell 144, 364–375. (doi:10.1016/j.cell.2011.01.008) 21277013 10.1016/j.cell.2011.01.008 PMC3089914

[67] van Breugel M et al. 2011 Structures of SAS-6 suggest its organization in centrioles. Science 331, 1196–1199. (doi:10.1126/science.1199325) 21273447 10.1126/science.1199325

[68] van Breugel M, Wilcken R, McLaughlin SH, Rutherford TJ, Johnson CM 2014 Structure of the SAS-6 cartwheel hub from Leishmania major. eLife 3, e01812 (doi:10.7554/eLife.01812) 24596152 10.7554/eLife.01812 PMC3939493

[69] Anderson RG, Brenner RM 1971 The formation of basal bodies (centrioles) in the Rhesus monkey oviduct. J. Cell Biol. 50, 10–34. (doi:10.1083/jcb.50.1.10) 4998200 10.1083/jcb.50.1.10 PMC2108422

[70] Fong CS, Kim M, Yang TT, Liao JC, Tsou MF 2014 SAS-6 assembly templated by the lumen of cartwheel-less centrioles precedes centriole duplication. Dev. Cell 30, 238–245. (doi:10.1016/j.devcel.2014.05.008) 25017693 10.1016/j.devcel.2014.05.008 PMC4116473

[71] Khodjakov A, Rieder CL, Sluder G, Cassels G, Sibon O, Wang CL 2002 De novo formation of centrosomes in vertebrate cells arrested during S phase. J. Cell Biol. 158, 1171–1181. (doi:10.1083/jcb.200205102) 12356862 10.1083/jcb.200205102 PMC2173237

[72] Szollosi D, Calarco P, Donahue RP 1972 Absence of centrioles in the first and second meiotic spindles of mouse oocytes. J. Cell Sci. 11, 521–541. 5076360 10.1242/jcs.11.2.521

[73] Vladar EK, Stearns T 2007 Molecular characterization of centriole assembly in ciliated epithelial cells. J. Cell Biol. 178, 31–42. (doi:10.1083/jcb.200703064) 17606865 10.1083/jcb.200703064 PMC2064416

[74] Guichard P, Desfosses A, Maheshwari A, Hachet V, Dietrich C, Brune A, Ishikawa T, Sachse C, Gonczy P 2012 Cartwheel architecture of *Trichonympha* basal body. Science 337, 553 (doi:10.1126/science.1222789) 22798403 10.1126/science.1222789

[75] Hirono M 2014 Cartwheel assembly. Phil. Trans. R. Soc. B 369, 20130458 (doi:10.1098/rstb.2013.0458) 25047612 10.1098/rstb.2013.0458 PMC4113102

[76] Jana SC, Marteil G, Bettencourt-Dias M 2014 Mapping molecules to structure: unveiling secrets of centriole and cilia assembly with near-atomic resolution. Curr. Opin. Cell Biol. 26, 96–106. (doi:10.1016/j.ceb.2013.12.001) 24529251 10.1016/j.ceb.2013.12.001

[77] Cottee MA, Raff JW, Lea SM, Roque H 2011 SAS-6 oligomerization: the key to the centriole? Nat. Chem. Biol. 7, 650–653. (doi:10.1038/nchembio.660) 21931303 10.1038/nchembio.660

[78] Hung LY, Tang CJ, Tang TK 2000 Protein 4.1 R-135 interacts with a novel centrosomal protein (CPAP) which is associated with the gamma-tubulin complex. Mol. Cell Biol. 20, 7813–7825. (doi:10.1128/MCB.20.20.7813-7825.2000) 11003675 10.1128/mcb.20.20.7813-7825.2000 PMC86375

[79] Basto R, Lau J, Vinogradova T, Gardiol A, Woods CG, Khodjakov A, Raff JW 2006 Flies without centrioles. Cell 125, 1375–1386. (doi:10.1016/j.cell.2006.05.025) 16814722 10.1016/j.cell.2006.05.025

[80] Cottee MA et al. 2013 Crystal structures of the CPAP/STIL complex reveal its role in centriole assembly and human microcephaly. eLife 2, e01071 (doi:10.7554/eLife.01071) 24052813 10.7554/eLife.01071 PMC3776556

[81] Hatzopoulos GN, Erat MC, Cutts E, Rogala KB, Slater LM, Stansfeld PJ, Vakonakis I 2013 Structural analysis of the G-box domain of the microcephaly protein CPAP suggests a role in centriole architecture. Structure 21, 2069–2077. (doi:10.1016/j.str.2013.08.019) 24076405 10.1016/j.str.2013.08.019 PMC3824074

[82] Zheng X et al. 2014 Conserved TCP domain of Sas-4/CPAP is essential for pericentriolar material tethering during centrosome biogenesis. Proc. Natl Acad. Sci. USA 111, E354–E363. (doi:10.1073/pnas.1317535111) 24385583 10.1073/pnas.1317535111 PMC3903230

[83] Matsuura K, Lefebvre PA, Kamiya R, Hirono M 2004 Bld10p, a novel protein essential for basal body assembly in *Chlamydomonas*: localization to the cartwheel, the first ninefold symmetrical structure appearing during assembly. J. Cell Biol. 165, 663–671. (doi:10.1083/jcb.200402022) 15173189 10.1083/jcb.200402022 PMC2172387

[84] Hiraki M, Nakazawa Y, Kamiya R, Hirono M 2007 Bld10p constitutes the cartwheel-spoke tip and stabilizes the 9-fold symmetry of the centriole. Curr. Biol. 17, 1778–1783. (doi:10.1016/j.cub.2007.09.021) 17900905 10.1016/j.cub.2007.09.021

[85] Roque H, Wainman A, Richens J, Kozyrska K, Franz A, Raff JW 2012 Drosophila Cep135/Bld10 maintains proper centriole structure but is dispensable for cartwheel formation. J. Cell Sci. 125, 5881–5886. (doi:10.1242/jcs.113506) 22976301 10.1242/jcs.113506

[86] Carvalho-Santos Z, Machado P, Branco P, Tavares-Cadete F, Rodrigues-Martins A, Pereira-Leal JB, Bettencourt-Dias M 2010 Stepwise evolution of the centriole-assembly pathway. J. Cell Sci. 123, 1414–1426. (doi:10.1242/jcs.064931) 20392737 10.1242/jcs.064931

[87] Mottier-Pavie V, Megraw TL 2009 Drosophila bld10 is a centriolar protein that regulates centriole, basal body, motile cilium assembly. Mol. Biol. Cell 20, 2605–2614. (doi:10.1091/mbc.E08-11-1115) 19321663 10.1091/mbc.E08-11-1115 PMC2682601

[88] Comartin D et al. 2013 CEP120 and SPICE1 cooperate with CPAP in centriole elongation. Curr. Biol. 23, 1360–1366. (doi:10.1016/j.cub.2013.06.002) 23810536 10.1016/j.cub.2013.06.002

[89] Mahjoub MR, Xie Z, Stearns T 2010 Cep120 is asymmetrically localized to the daughter centriole and is essential for centriole assembly. J. Cell Biol. 191, 331–346. (doi:10.1083/jcb.201003009) 20956381 10.1083/jcb.201003009 PMC2958470

[90] Lin YN, Wu CT, Lin YC, Hsu WB, Tang CJ, Chang CW, Tang TK 2013 CEP120 interacts with CPAP and positively regulates centriole elongation. J. Cell Biol. 202, 211–219. (doi:10.1083/jcb.201212060) 23857771 10.1083/jcb.201212060 PMC3718976

[91] Keller LC, Romijn EP, Zamora I, Yates JR 3rd, Marshall WF 2005 Proteomic analysis of isolated chlamydomonas centrioles reveals orthologs of ciliary-disease genes. Curr. Biol. 15, 1090–1098. (doi:10.1016/j.cub.2005.05.024) 15964273 10.1016/j.cub.2005.05.024

[92] Fourrage C, Chevalier S, Houliston E 2010 A highly conserved Poc1 protein characterized in embryos of the hydrozoan *Clytia hemisphaerica*: localization and functional studies. PLoS ONE 5, e13994 (doi:10.1371/journal.pone.0013994) 21103375 10.1371/journal.pone.0013994 PMC2982836

[93] Keller LC, Geimer S, Romijn E, Yates J 3rd, Zamora I, Marshall WF 2009 Molecular architecture of the centriole proteome: the conserved WD40 domain protein POC1 is required for centriole duplication and length control. Mol. Biol. Cell 20, 1150–1166. (doi:10.1091/mbc.E08-06-0619) 19109428 10.1091/mbc.E08-06-0619 PMC2642750

[94] Pearson CG, Osborn DP, Giddings TH Jr, Beales PL, Winey M 2009 Basal body stability and ciliogenesis requires the conserved component Poc1. J. Cell Biol. 187, 905–920. (doi:10.1083/jcb.200908019) 20008567 10.1083/jcb.200908019 PMC2806327

[95] Venoux M, Tait X, Hames RS, Straatman KR, Woodland HR, Fry AM 2013 Poc1A and Poc1B act together in human cells to ensure centriole integrity. J. Cell Sci. 126, 163–175. (doi:10.1242/jcs.111203) 23015594 10.1242/jcs.111203 PMC3603514

[96] Spektor A, Tsang WY, Khoo D, Dynlacht BD 2007 Cep97 and CP110 suppress a cilia assembly program. Cell 130, 678–690. (doi:10.1016/j.cell.2007.06.027) 17719545 10.1016/j.cell.2007.06.027

[97] Graser S, Stierhof YD, Lavoie SB, Gassner OS, Lamla S, Le Clech M, Nigg EA 2007 Cep164, a novel centriole appendage protein required for primary cilium formation. J. Cell Biol. 179, 321–330. (doi:10.1083/jcb.200707181) 17954613 10.1083/jcb.200707181 PMC2064767

[98] Sillibourne JE, Specht CG, Izeddin I, Hurbain I, Tran P, Triller A, Darzacq X, Dahan M, Bornens M 2011 Assessing the localization of centrosomal proteins by PALM/STORM nanoscopy. Cytoskeleton (Hoboken) 68, 619–627. (doi:10.1002/cm.20536) 21976302 10.1002/cm.20536

[99] Tanos BE, Yang HJ, Soni R, Wang WJ, Macaluso FP, Asara JM, Tsou MF 2013 Centriole distal appendages promote membrane docking, leading to cilia initiation. Genes Dev. 27, 163–168. (doi:10.1101/gad.207043.112) 23348840 10.1101/gad.207043.112 PMC3566309

[100] Jerka-Dziadosz M, Gogendeau D, Klotz C, Cohen J, Beisson J, Koll F 2010 Basal body duplication in Paramecium: the key role of Bld10 in assembly and stability of the cartwheel. Cytoskeleton (Hoboken) 67, 161–171. (doi:10.1002/cm.20433). 20217679 10.1002/cm.20433

[101] Ohta T, Essner R, Ryu JH, Palazzo RE, Uetake Y, Kuriyama R 2002 Characterization of Cep135, a novel coiled-coil centrosomal protein involved in microtubule organization in mammalian cells. J. Cell Biol. 156, 87–99. (doi:10.1083/jcb.200108088) 11781336 10.1083/jcb.200108088 PMC2173569

[102] Carvalho-Santos Z et al. 2012 BLD10/CEP135 is a microtubule-associated protein that controls the formation of the flagellum central microtubule pair. Dev. Cell 23, 412–424. (doi:10.1016/j.devcel.2012.06.001) 22898782 10.1016/j.devcel.2012.06.001

[103] Chen Z, Indjeian VB, McManus M, Wang L, Dynlacht BD 2002 CP110, a cell cycle-dependent CDK substrate, regulates centrosome duplication in human cells. Dev. Cell 3, 339–350. (doi:10.1016/S1534-5807(02)00258-7) 12361598 10.1016/s1534-5807(02)00258-7

[104] Kohlmaier G et al. 2009 Overly long centrioles and defective cell division upon excess of the SAS-4-related protein CPAP. Curr. Biol. 19, 1012–1018. (doi:10.1016/j.cub.2009.05.018) 19481460 10.1016/j.cub.2009.05.018 PMC2993638

[105] Schmidt TI, Kleylein-Sohn J, Westendorf J, Le Clech M, Lavoie SB, Stierhof YD, Nigg EA 2009 Control of centriole length by CPAP and CP110. Curr. Biol. 19, 1005–1011. (doi:10.1016/j.cub.2009.05.016) 19481458 10.1016/j.cub.2009.05.016

[106] Arquint C, Gabryjonczyk AM, Imseng S, Bohm R, Sauer E, Hiller S, Nigg EA, Maier T 2015 STIL binding to Polo-box 3 of PLK4 regulates centriole duplication. eLife. 4 (doi:10.7554/eLife.07888) 10.7554/eLife.07888 PMC4530586 26188084

